# On the binding affinity of macromolecular interactions: daring to ask why proteins interact

**DOI:** 10.1098/rsif.2012.0835

**Published:** 2013-02-06

**Authors:** Panagiotis L. Kastritis, Alexandre M. J. J. Bonvin

**Affiliations:** Bijvoet Center for Biomolecular Research, Faculty of Science, Chemistry, Utrecht University, Padualaan 8, 3584 CH Utrecht, The Netherlands

**Keywords:** dissociation constant, protein interaction models, protein complex modelling, protein–protein docking, scoring functions, structure–affinity relations

## Abstract

Interactions between proteins are orchestrated in a precise and time-dependent manner, underlying cellular function. The binding affinity, defined as the strength of these interactions, is translated into physico-chemical terms in the dissociation constant (*K*_d_), the latter being an experimental measure that determines whether an interaction will be formed in solution or not. Predicting binding affinity from structural models has been a matter of active research for more than 40 years because of its fundamental role in drug development. However, all available approaches are incapable of predicting the binding affinity of protein–protein complexes from coordinates alone. Here, we examine both theoretical and experimental limitations that complicate the derivation of structure–affinity relationships. Most work so far has concentrated on binary interactions. Systems of increased complexity are far from being understood. The main physico-chemical measure that relates to binding affinity is the buried surface area, but it does not hold for flexible complexes. For the latter, there must be a significant entropic contribution that will have to be approximated in the future. We foresee that any theoretical modelling of these interactions will have to follow an integrative approach considering the biology, chemistry and physics that underlie protein–protein recognition.

## 1. Historical perspective

In order to understand our current view of proteins and their interactions, one has to understand how previous knowledge about proteins was accumulated. The present work rests on the shoulders of our predecessors, who essentially determined the route of protein research in today's post-genomic era. It is truly amazing that we are able to routinely characterize and understand protein folding, dynamics and interactions to such an extent and at such detailed resolution. How did we end up with such a vast amount of data for protein molecules? Protein science is exactly 223 years old, which translates into 224 years of trying to understand the nature of protein molecules.

Antoine François, comte de Fourcroy (1755–1809), successfully distinguished several types of proteins back in 1789, including albumin, fibrin, gelatin and gluten. Some years later, Jöns Jacob Berzelius (1779–1848), in a letter to Gerardus Johannes Mulder (1802–1880) dated 10 July 1838, first suggested the term protein to describe a distinct class of biomolecules, stating:

The name protein that I propose for the organic oxide of fibrin and albumin, I wanted to derive from [the Greek word] πρωτ*ɛι̃*ος, because it appears to be the primitive or principal substance of animal nutrition.

While at Utrecht University, The Netherlands, Mulder described the chemical composition of fibrin, egg albumin and serum albumin [1], which was pioneering work that led to the initial and critical observation that distinct proteins are composed of the same chemical elements: carbon, nitrogen, oxygen, hydrogen, phosphorus and sulphur. Additionally, Mulder successfully characterized protein degradation products, such as leucine, determining an approximately correct molecular weight of the residue (131 Da) [2].

In 1902, Franz Hofmeister (1850–1922) and Emil Fischer (1852–1919), who spoke at a meeting in Karlsbad shortly after one another, independently announced that proteins are linear polymers consisting of amino acids linked by peptide bonds. The nature of the peptide bond in addition to the successful synthesis of the first optically active peptides by Otto Warburg in Fischer's laboratory were greatly influenced by the search for the 20 building blocks of proteins and prompted the investigation of the last few that were by that time still unknown: amino acid residues were recognized as protein constituents based on isolation from protein hydrolysates in a timeline of approximately 130 years [3] (leucine being the first, identified in 1819 [4], and threonine being the last, identified in 1936 [5]). The primary structure of the proteins was finally elucidated in 1949, when Fred Sanger sequenced bovine insulin [6].

In the late 1950s, John Kendrew determined the first crystal structure, that of sperm whale myoglobin [7], whereas Max Perutz determined the crystal structure of haemoglobin [8]. Both Kendrew and Perutz were protagonists in a blossoming era for X-ray crystallography, working closely together with William and Laurence Bragg, William Astbury and John Desmond Bernal. Interestingly, the crystal structure of haemoglobin is composed of four subunits, all non-covalently bound. Such a quarternary structure did not come as a surprise, since Theodor Svedberg had already determined the molecular weight of haemoglobin and, therefore, its subunit composition in the mid-1920s [9]. Therefore, one should not forget that the discovery of the quaternary structure (QS) preceded the discovery of the primary [6], secondary [10,11] and tertiary structures of proteins [7,8].

Whereas X-ray crystallography has proven to be the primary method for studying the atomic structure of biological macromolecules, nuclear magnetic resonance (NMR) spectroscopy allows both the three-dimensional structure and the dynamics of biomacromolecules to be probed. Kurt Wüthrich with his group outlined a framework for NMR structure determination of proteins in 1982 [12]. Two years later, the first de novo NMR structure of a protein in solution was determined—that of the bull seminal protease inhibitor [13], reported the same year as the Lac repressor headpiece [14]. In the following years, structures of a plethora of biomacromolecules have been determined by X-ray crystallography and NMR and, as of November 2012, approximately 87 000 structures have been deposited in the public repository of macromolecular structures, the Protein Data Bank (PDB database) [15,16].

Although the PDB already includes thousands of macromolecular complexes involved in protein–protein interactions, their importance in defining and orchestrating cellular processes was only recently appreciated [17,18]. A partial explanation could be that the Aristotelian concept of life that ‘the whole is greater than the sum of its parts’, erroneously considered as the central dogma of vitalism, seemingly contradicted the already established mechanistic view of molecular biology.

In the case of protein synthesis, it was known that macromolecular interactions must play a major role. Still, DNA replication, transcription and translation were unexplored areas in biology at that time and up to now have been considered active areas of research. On the other hand, complete metabolic processes were characterized in detail, such as glycolysis [19], the Krebs cycle [20], cholesterol and fatty acid biosynthesis [21], which, again, erroneously led the community to believe that interactions were not essentially involved in the cellular metabolism. Subsequently, the dogma ‘one gene/one enzyme/one function’, framed by Beadle and Tatum [22], was being validated, stating that simple, linear connections are expected between the genotype and the phenotype of an organism. Therefore, up to the 1970s, macromolecular interactions were considered purification artefacts. For example, during the isolation and characterization of enzymes *in vitro*, several experimental difficulties arose as a result of protein–protein interactions, such as co-precipitation, which was believed to be contamination [23].

However, a unique observation back in 1958 by Frederic Richards gradually started to spark the interest in protein interaction phenomena [24]: Richards found that RNase A resulted in a cleaved product, RNase S, when a particular protease was used (subtilisin). RNase S is composed of two molecules, the S-peptide and the S-protein. When these are separated, no RNase activity is observed; however, when recombined in the test tube, the RNase activity is recovered [24]. Richards also foresaw the importance of the interactions of colicin molecules with their macromolecular substrates [25] and laid the foundations for the analysis of macromolecular interactions by implementing the well-known Lee & Richard's [26] algorithm for calculating accessible surface areas of biomolecules. In 1974, Robert Huber's group elucidated the crystal structure of the first protein–inhibitor complex [27]—that of bovine trypsin with its pancreatic trypsin inhibitor. Cyrus Chothia and Joël Janin [28] first characterized the structure and stability factors of the formed interface and concluded that the intrinsic interaction energy was simply proportional to the area of the interface, a first, rather coarse, but critical approximation to understand protein–protein binding. A few years later, in 1978, Shoshana Wodak and Joël Janin [29] implemented the first modelling algorithm for docking protein molecules.

In the following years, an increasing amount of data for protein–protein interactions was accumulated and dogmas about single protein function were being scrambled one by one: For DNA replication, which was thought to be catalysed by a single molecule in the 1960s [30], the involvement of other proteins (e.g. DNA helicase, DNA primase, single-strand binding proteins) was found to be essential for fulfilling this task apart from the polymerase [31]. For protein transport to the mitochondria, more than 20 proteins were identified as critical for this process [32]. In a meeting review published in *Cell* in 1992, Bruce Alberts & Miake-Lye stated that:

… cell biochemistry would appear to be largely run by a set of protein complexes, rather than proteins that act individually and exist in isolated species.

**Figure 1.**
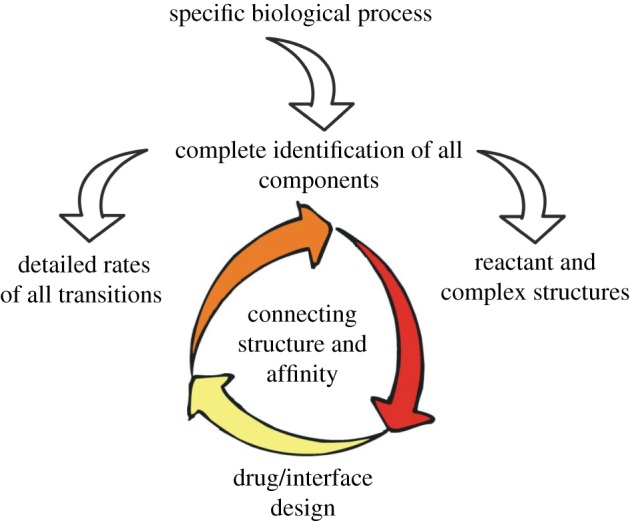
Methodology to follow in protein–protein interaction identification leading to drug/interface design.

Consequently, to understand how the cell works, a holistic approach needs to be followed (shown in figure 1). Over the last 20 years, this approach has yielded on a daily basis fascinating results in both fundamental [33–39] and applied [40–43] research. The outcome is substantial not only for understanding life at the cellular level, but also for drug design: dissection of protein–protein interactions has opened routes to the production of therapeutics with novel functions aiming to cure, for example, amyloidosis-related diseases [44,45] and cancer [46,47].

## 2. Role of protein quaternary structure in a cell

The levels of protein structure were first portrayed by Linderstrøm-Lang & Schnellman [48], which defined QS as being the highest level of structural hierarchy described by the interactions of two or more non-covalently bound subunits that eventually form a functional molecule. QS was first used to designate obligate complexes, such as haemoglobin [8], and its main difference from non-obligate complexes lies in the nature of the interacting subunits: if the individual components of a complex can exist free in solution, then the complex is non-obligate; in contrast, if these subunits constitute an integral part of the structure and cannot be separated (or, if separated, the structure and function of the protein is irreversibly lost) then the complex is referred to as being obligate. Note that the definition of non-obligate and obligate interactions can also depend on the localization (for details, see §5.1).

Several sections in collective books [49–51], original publications [52] and critical reviews [53–58] have concentrated on describing the nature of both obligate and non-obligate interactions, whereas, more recently, reviews about the structure, function and modulation of non-obligate complexes have also appeared [59–62].

In this review, the focus will be on describing the structure and function of non-obligate protein–protein complexes in the context of recent findings, explaining the underlying theory of how and why proteins interact as well as the recently accumulated knowledge for their underlying affinity, describing the efforts to connect QS to binding affinity.

Along with the description of recent findings, fundamental past observations will be assessed and a critical view on modern models will be posed. The main motivation behind this is the central role that protein–protein interactions play in defining the fundamental functional and structural unit of all living matter, the cell. Since the biological function of a protein is defined by its interactions in the cell [63] and inappropriate interactions can lead to diseases such as amyloidoses [44,45] and cancer [46,47], development of methods aiming to disrupt or modulate protein–protein interactions is critical [64]. Therefore, in order to successfully design drugs or interfaces with predefined properties, knowledge and understanding of binding affinity and its underlying contributing factors is deemed mandatory.

### 2.1. Determination of non-obligate quaternary structure at atomic resolution: how do proteins interact?

A plethora of non-obligate protein–protein complexes have been successfully determined using traditional techniques, such as X-ray crystallography and NMR spectroscopy. These techniques provide a detailed picture of how proteins interact at atomic resolution, meaning that their interfaces (defined as the regions involved in protein interactions) are well characterized and the contributing interactions documented. For example, water molecules important for the interaction can be described, as well as formed salt bridges, hydrogen bonds, degree of complementarity of the two partners directly linked with the strength of the van der Waals interactions, etc. Also, the shape of the interface can be examined and classified as being concave or convex, whereas the biochemical nature of the interface and the rim (the area in its close vicinity) is recognized by observing the contributing amino acid residues. Such analysis is trivial and very frequently used to compare properties of complexes of a different nature [65–69]. Despite that, it has been argued that the sizes of the datasets of derived protein–protein complexes have often been too small, which may lead to statistically unreliable conclusions [70]. Several tools of central importance are routinely used that are able to recognize structural parameters for protein–protein complexes [71], including NACCESS [26] for surface calculations and HBPLUS [72] for recognizing water molecules at the interface and the underlying contacts. Several webservers have also been designed to aid the annotation of macromolecular interfaces [73–77], such as PISA [74] (http://www.ebi.ac.uk/msd-srv/prot_int/), and comprehensive databases compiled, such as PICCOLO [77] (http://www-cryst.bioc.cam.ac.uk/databases/piccolo). Recognizing the interfacial region is of particular importance in protein–protein complexes since the biological function of the complex is in most cases directly related to the interactions made [78].

### 2.2. The concept of buried surface area and its inherent limitations

In protein–protein interactions, the buried surface area (BSA) is defined as the surface buried away from the solvent when two or more proteins or subunits associate to form a complex. The most widely used surface calculation method is the solvent-accessible surface introduced by Lee & Richards [26]. In this method, a probe sphere traces the solvent-accessible surface as it rolls over the protein. Protein atoms are assigned their corresponding van der Waals radii. The solvent-accessible surface area traced by the centre of the sphere can be considered as an expanded van der Waals surface of the molecule. In another method, if a water-sized probe sphere touches the protein surface, then this surface is defined as the contact surface (i.e. the contact point instead of the centre of the sphere is used to trace the surface). Since different methods have been developed to calculate and represent the protein surface to date [79–83], the area calculated is clearly dependent on both the method used and the radii considered for the protein atoms and the probe sphere. For example, different van der Waals radii have been reported for atoms in biomacromolecules [84] and substantial differences in the algorithms used to calculate and represent molecular surfaces have been noted by Michael Connolly (http://www.netsci.org/Science/Compchem/feature14.html).

Besides that, another inherent limitation for the calculation of BSAs of protein–protein complexes lies in the fact that proteins do not associate as rigid entities, but may undergo small-to-large conformational changes upon binding. Therefore, in order to calculate BSA one has to know in detail the three-dimensional structures of the unbound states of the proteins that interact, and calculate the BSA according to2.1
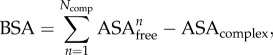
where 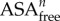 indicates the accessible surface area of the unbound molecules and ASA_complex_ that of the bound complex.2.2
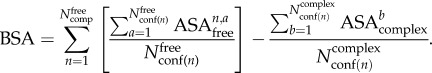


However, since proteins undergo dynamic motions directly associated with their function [85] the surface area that is calculated using (2.1) represents an approximate value and not necessarily the expanded van der Waals surface that should be averaged over the surface formed by all conformations of the free reactants (assuming that conformations of the reactants are equally populated for simplification) and the bound structure: where *N*_comp_ indicates the total number of free components in the complex, *a* all possible representative conformations 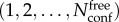 of the free reactant *n*, and *b* all possible representative conformations 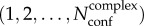 of the complex.

However, although equation (2.2) is analytical, for simplification purposes, equation (2.1) is used. Hence, in BSA calculations, proteins are currently considered static and, when the unbound structures are not available, the accessible surface area is calculated from the separated components of the complex, therefore considering that proteins bind as rigid bodies. An interesting question about the definition of the functional surface of protein–protein interactions is whether functional solvent molecules or interacting ions and cofactors should be included in the calculations, since solvent has been proposed to functionally define the protein structure [86,87].

### 2.3. Non-covalent interactions formed in the interface and accepted approximations

**Figure 2.**
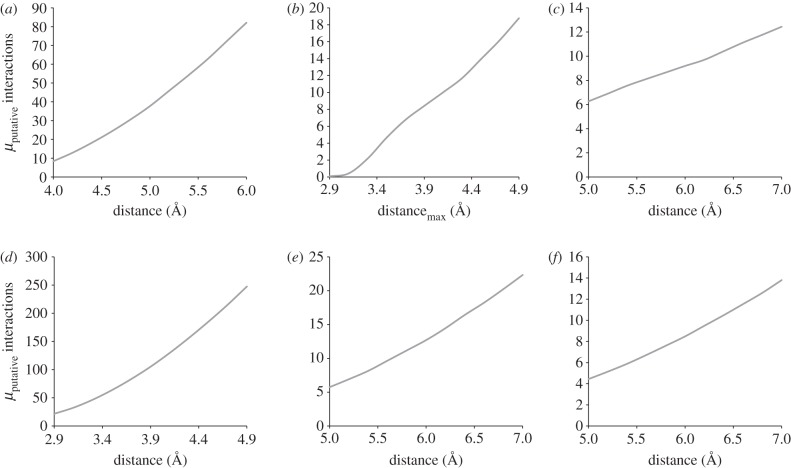
Change in the number of intermolecular interactions for 195 protein–protein complexes using cut-offs ±1 Å. *μ* corresponds to the average value calculated. (*a*) Hydrophobic contacts, (*b*) hydrogen bonds, (*c*) ionic, (*d*) van der Waals, (*e*) aromatic and (*f*) *π*–cation interactions.

During the study of the three-dimensional structure of a macromolecular complex in its bound conformation, molecular interactions present in the interface can be annotated. This annotation is an integral part of any structural analysis of a derived complex and has been recently critically reviewed [88]. One of the major inconsistencies found in the literature is the usage of different cut-offs for inter-residue interactions ranging from 5 to 14 Å [89–92]. Because of this, there is no consensus on the geometrical definition of non-covalent interactions [93–95]. Deviations in the cut-offs for specific interactions can also be found in the literature. Furthermore, hydrophobic contacts can be analysed via a residue-based criterion (e.g. using the Kyte–Doolittle scale [96]) or an atom-based criterion, where hydrophobic contacts are defined between atoms within 5 Å from each other [77]. The distance between a donor and an acceptor atom to define a hydrogen bond also varies slightly between various web servers [74–77]. Other interactions, such as annotation of aromatic–sulphur or aromatic–aromatic interactions also follow different criteria [76,77] depending on the method used [97–101]. As a consequence, the different cut-offs used for analysing crystal structures hamper a direct comparison of annotated intermolecular interactions in the literature in a large-scale manner. Figure 2 illustrates how the number of interactions found for 195 protein–protein complexes [102,103] substantially changes by varying the cut-off by ±1 Å [77]: their number changes as a function of distance in a, not entirely, linear manner. This also indicates that the number of interactions cannot simply be related to the binding strength and used to classify complexes as strong or weak binding, as also highlighted previously [102].

#### 2.3.1. Considerations for solvent effects

Since the release of the first crystal structure of a heteromeric complex [27]—that of trypsin with the pancreatic trypsin inhibitor (PTI)—the role of water has been clearly demonstrated: the side chain of Asp^189^ of trypsin is in contact with the Lys^15^ side chain of PTI via water-mediated hydrogen bonds. Its importance is also highlighted in the structure of trypsin in complex with the homologous inhibitor from soybean (STI), where the water molecule is absent, since the salt bridge is formed directly via the bulkier positively charged residue Arg of STI that substitutes Lys^15^. Apart from crystallography, various methods [104] can tackle not only the structure but also the dynamics of water molecules at protein surfaces and at interfaces of protein–protein complexes such as high-resolution neutron diffraction and multi-dimensional NMR. For example, buried water molecules for PTI observed in solution by NMR are in excellent agreement with crystallographic data [105].

**Figure 3.**
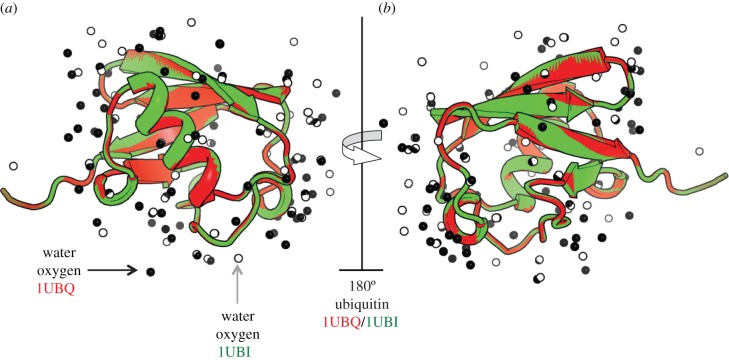
(*a*,*b*) Crystallographically determined structures of ubiquitin (PDB entries 1UBQ and 1UBI), along with their corresponding crystallographic water molecules. Ubiquitin is shown in cartoon representation, whereas the oxygen atoms of water are shown as spheres.

Recently, several experimental [106] and theoretical [107] advances have provided deeper understanding in the structure of water around biomolecules. However, inconsistencies between the long-lived residence time of water molecules measured in solution and the NMR structures and positions of water molecules observed in protein crystals still exist [106]. Differences in water structure can even be seen between crystal structures of the same resolution (1.8 Å) and same space group (figure 3*a*,*b*). In a recent study, it was shown that the appearance of a catalytic water molecule in the electron density obtained by X-ray diffraction depends on whether the structure was determined under cryo- or ambient conditions [108].

Water molecules in the interface of protein–protein complexes may have structural and/or functional roles, depending on their interactions [86,109]. For example, water-mediated hydrogen bonds in an interface can contribute significantly to binding [110–112]. Water buried in the interface, filling interfacial ‘gaps’, has also been frequently reported [86], having an ambiguous role in modulating interfacial properties, since only a few H-bonds are formed and van der Waals interactions seem to dominate [112]. Interfacial water often participates in extensive water networks [113]; the latter have been observed in highly solvated interfaces, such as in those of colicins in complex with their cognate or non-cognate immunity proteins [114] and in the barstar inhibitor barnase in complex with its cognate and non-cognate partner, barstar [115] and RNAse S1 [116], respectively.

Water can also participate in allosteric phenomena [117]. Royer *et al.* [117] established that interfacial water of the dimeric haemoglobin from *Scapharca inaequivalvis* is modulating the molecule's allosteric cooperativity and contributes to fast communication between the subunits via vibrational energy transport that occurs on the 1–10 ps time scale [118]. Even in the self-assembly of amyloid fibrils, water is being considered as an active component in the process defining different interaction pathways [119]. One-dimensional water wires at the interface of polar amyloidogenic proteins that are gradually expelled mediate the interaction of the forming fibrils [119], whereas, for hydrophobic peptides, the assembly of the two sheets and expulsion of water molecules occur nearly simultaneously [119]. Hydrophobic surfaces bind much faster (nearly 1000-fold) than hydrophilic ones, since trapped water creates a barrier to rapid assembly.

In order to obtain biophysical insights into the role of water in protein–protein interactions during the association process, most theoretical studies on protein folding and association deal mostly with hydrophobic interfaces [120,121], showing that hydrophobic dewetting is fundamental for the interaction. Yet, dewetting must occur rarely *in vitro* and *in vivo* since few polar residues are enough to prevent the phenomenon [122]. On average, for protein–protein complexes approximately 70 per cent of the interfacial residues are hydrophilic.

The association mechanism of hydrophilic interfaces has only recently been investigated [113], showing that interfacial water may form an adhesive hydrogen-bond network between the interfaces at the encounter complex stage of association and consequently stabilize early intermediates before native contacts are formed. Note that this does not contradict Janin's observations for the percentage of hydration of protein–protein interfaces, which is around 25 per cent [66], since only a few residues will retain their water molecules in the product complex; the others will form hydrogen bonds and salt bridges with other polar residues and/or backbone atoms.

Overall, in years to come, the advent of both experimental and computational techniques to map the structure, position and dynamics of water molecules around proteins will allow the study of water–protein interactions in a more detailed manner, unveiling fundamental roles for water, currently either hypothesized or even unknown [86,109,122], and this in much more complicated environments, such as that of the cell itself [109,123].

## 3. Definition of binding affinity for macromolecular recognition

The binding of two proteins can be viewed as a reversible and rapid process in an equilibrium that is governed by the law of mass action. The binding affinity is the strength of the interaction between two (or more than two) molecules that bind reversibly (interact). It is translated into physico-chemical terms in the dissociation constant (*K*_d_), the latter being the concentration of the free protein that occupies half of the overall sites of the second protein at equilibrium.

By convention, the protein present in fixed and limited amounts will be termed the receptor protein (A), whereas the reaction component that is varied will be termed the ligand protein (B).

Certain assumptions inherent to any measurement of a protein–protein interaction should be considered:

— All interactions studied are assumed to be reversible and the association reaction is bimolecular; on the other hand, the dissociation reaction is unimolecular.
— The receptor protein must have a fixed concentration and, therefore, receptor molecules are equivalent and independent (do not interact).
— The interactions are measured at equilibrium.
— The two proteins that are measured in solution do not undergo any other chemical reactions and are assumed to exist only in their free or bound states.
— The measured affinity (*K*_d_) is proportional to the number of occupied receptor binding sites.

Therefore, for a simple reversible reaction between proteins A and B, one can write:3.1
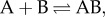
and, in more detail,3.2
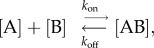
where [A] and [B] denote the concentrations of the free proteins (reactants), whereas [AB] denotes the concentration of their bound complex (product). *k*_on_ represents the association rate constant, measured in M^−1^s^−1^; *k*_off_ represents the dissociation rate constant.

When the system is at equilibrium, *K*_d_ is defined as3.3
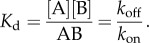


One can re-write equation (3.2) in terms of total concentration of both proteins [A] and [B]. After applying the assumption for the conservation of mass, where3.4
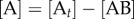
and3.5
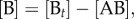
and introducing these in equation (3.3), one gets3.6
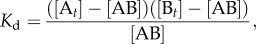
and, by re-arranging equation (3.6), this gives the fractional saturation (FS)3.7
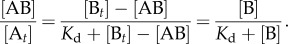


In other words, and according to equation (3.7), the FS corresponds to the fraction of the molecules of protein A that are saturated with the molecules of protein B.

By assuming that a single binding site is present, a rectangular hyperbola will be visible in a plot of FS [AB]/[A*_t_*] versus [B]. Instead, one might highlight these binding events using a plot of FS [AB]/[A*_t_*] versus log[B], or the well-known Scatchard plot, a plot of ligand bound/ligand free.

The Scatchard plot is the traditional method for analysing binding data where the concentration of the ligand [B] is measured. It is described by the following equation:3.8
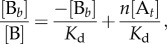
where a straight line is derived for the simple model (one binding site is present) and *n* denotes the stoichiometry of the interaction (in the simple case, *n* = 1) and [B*_b_*] the concentration of the bound ligand. The straight line's characteristics are: *x*-intercept, *n*[A*_t_*]; *y*-intercept, *n*[A*_t_*]/*K*_d_; slope, −1/*K*_d_.

**Figure 4.**
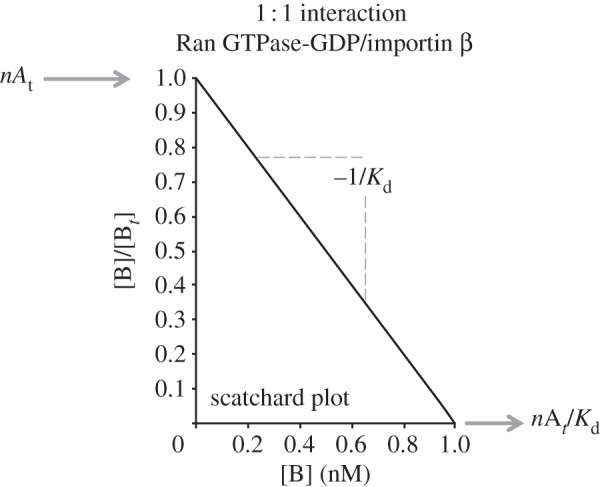
Simulated scatchard plot for Ran GTPase-GDP and importin β. We assume a 1 : 1 interaction, having exactly 1 nM affinity (see text).

As an example, a simulated Scatchard plot for the 1 nM interaction between Ran GTPase–GDP and importin *β* is illustrated in figure 4, showing the abovementioned characteristics.

It is quite useful to assess the linearity of the Scatchard plot, since deviation from simple binding (and, therefore, distortion of the linearity of the plot) is expected to be the result of either multiple sites or non-specific binding, which may be difficult to distinguish in practice [124].

The binding affinity can also be translated in physical terms into the Gibbs free energy of dissociation (*ΔG*_d_), which, for an interaction to occur, must be positive,3.9
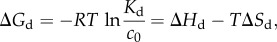
where *c*_0_ is the concentration that defines the standard state, being 1 mol l^−1^ by conventional criteria, *R* is the gas constant (8.3144 J K^−1^ mol^−1^ equal to 1.9872 cal K^−1^ mol^−1^), *T* is the absolute temperature (kelvin), whereas *ΔH*_d_, *ΔS*_d_ and *ΔG*_d_ denote, respectively, the changes in enthalpy, entropy and binding free energy upon complex dissociation. The binding affinity is related to the Gibbs free energy of association (*ΔG*_a_) as3.10
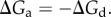


Both free energies describe all the chemical and energetic factors involved in the dissociation and association reaction, respectively.

The free energy of binding, *ΔG*_a_, can be decomposed into two opposing general energies, one favouring the complexation of the unbound partners and one opposing it,3.11
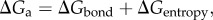
where *ΔG*_bond_ and *ΔG*_entropy_ denote the intrinsic ‘non-bonded interaction energy’ that includes all chemical forces acting on the interface of the complex and entropy, respectively, analogous to the physical enthalpy and entropy changes, respectively. Such simplification is useful for assessing the energy of macromolecular binding and has been rediscovered several times [28,125,126], from recognizing forces that participate in insulin dimerization [125] to analysis of cooperative effects of protein–protein interactions [127].

### 3.1. Experimental methods and associated errors

Understanding complex biochemical pathways requires quantitative *in vitro* analysis of protein–protein binding [128–130]. For the determination of the FS or binding parameters of a biological reaction between two proteins in such pathways, several methods have been developed [131,132], including NMR spectroscopy, equilibrium dialysis, dynamic light scattering, analytical ultracentrifugation, ultrafiltration, electrophoretic methods, differential scanning calorimetry, homogeneous time-resolved fluorescence, fluorescence correlation spectroscopy/fluorescence cross-correlation spectroscopy, spectroscopic assays, affinity capillary electrophoresis, biolayer interferometry, dual polarization interferometry, static light scattering and microscale thermophoresis. Overall, these methods can be classified in two general categories, namely direct (or separative) and indirect (non-separative) methods [133]. Direct methods measure the actual concentrations of the bound and free proteins, whereas indirect methods imply the concentrations from a signal that is being observed.

Gel filtration, ultracentrifugation, ultrafiltration or equilibrium dialysis are direct methods that can be used to measure binding of protein–protein interactions. Direct methods might be appropriate only for binding reactions exhibiting slow dissociation rates, since the process of separating the bound and free proteins must be faster than the rate of dissociation of the complex. If dissociation and separation of the reactants occur on similar time scales, these methods are inappropriate since the equilibrium will be disturbed by the separation of the reactants [133].

Optical methods, such as absorbance, resonance or fluorescence spectroscopy techniques, belong to the indirect methods, where the assumption is made that the measured signal is directly proportional to the concentration of the product, assuming that the proteins exist in only two states: the free and the bound populations, with each having its unique optical characteristic. Consequently, if O_B_ is the signal when protein B is present at a given concentration, O_0_ the signal in its absence, and O_sat_ the value at saturation of the reaction, one can measure the FS using3.12
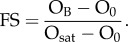


Three of the most frequently used methods to measure the binding affinity of protein–protein interactions will be compared and discussed in more detail in the following, namely isothermal titration calorimetry (ITC) [134], surface plasmon resonance (SPR) [135] and fluorescence-based methods [136]. One should, however, bear in mind that more than 20 methods have been described in the literature for determining biomolecular binding kinetics [137]. The determination of the actual affinity clearly depends on the method used along with its inherent sensitivity and on the strength of the interactions that are being measured.

#### 3.1.1. Isothermal titration calorimetry

**Figure 5.**
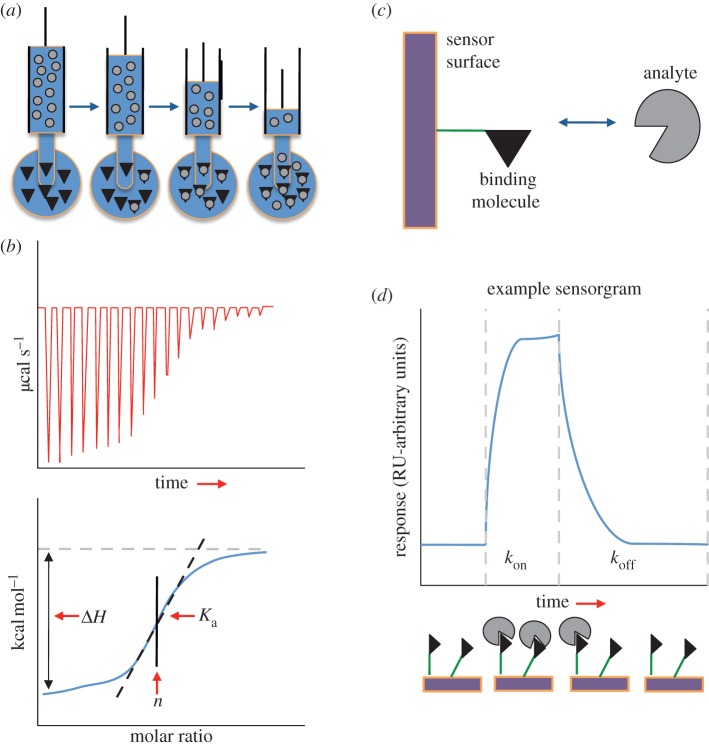
(*a,b*) Isothermal titration calorimetry and (*c,d*) surface plasmon resonance (SPR) techniques. (*a*) Titrations used to measure heat capacity changes and (*b*) calculation of *K*_a_. (*c*) SPR method and (*d*) monitoring of the association/dissociation process of the mobile agent. See text for details.

One of the most commonly used calorimetric approaches to study protein–protein interactions is ITC, which measures the heat uptake or release during a biomolecular interaction. An ITC experiment consists of successive additions of protein B to a solution of protein A, the latter contained in a reaction cell (figure 5*a*). Each addition leads to a specific amount of protein–protein complexes, as dictated by the binding affinity that can be observed by monitoring the heat release (or uptake; figure 5*b*).

Microcalorimetry reports on the enthalpy of association, *ΔH*_a_, that can be related directly to the dissociation enthalpy, *ΔH*_d_; if the titration is performed at different temperatures, changes in heat capacity (*ΔC*_p_) at constant pressure are also reported and are equal to3.13
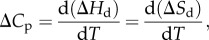
where d*T* corresponds to the changes in the temperature.

What distinguishes ITC from the other techniques is that, besides measuring binding affinity, it also allows the enthalpy, entropy and change in heat capacity of the interaction (*ΔH*_d_, *ΔS*_d_ and *ΔC*_p_, respectively) to be determined. On the other hand, ITC cannot be used for very low- or very high-affinity protein–protein interactions since the change in heat capacity is not correctly captured by the method. However, some studies have reported affinity data obtained with ITC for very low-affinity complexes [102].

#### 3.1.2. Surface plasmon resonance

SPR is an optical method to measure the refractive index near a sensor surface. In Biacore, particularly, this surface forms the floor of a flow cell through which an aqueous solution can pass under continuous flow (figure 5*c*). In order to detect a binary interaction, one protein is immobilized onto the sensor surface. Its binding partner (the analyte) is injected into the aqueous solution through the flow cell. As the analyte binds to the immobilized partner, the accumulation of proteins on the surface results in an increase in the refractive index. Measurement of this change is performed and the result is plotted as response units (RUs) versus time (figure 5*d*). After a defined association time, a solution without the analyte is injected that dissociates the bound complex between the immobilized protein and the partner. During dissociation, a decrease in SPR signal (expressed in RUs) is observed. From these, kinetic constants can be retrieved; however, one should keep in mind that protein immobilization affects the conformational and rotational entropy, and, therefore, association rates. On the other hand, SPR has been shown to be the preferred method for characterizing the kinetics for protein–protein interactions, since most reported *K*_d_s are determined by this method [136]. However, since diffusion is affected when using SPR, other methods should be used for *k*_on_ data collection [138].

#### 3.1.3. Fluorescence-based methods

In most of these methods (e.g. fluorescence (de)polarization (FP) or Förster resonance energy transfer, competitive binding assays are used in which a labelled ligand molecule is bound and subsequently displaced by any of a variety of competitive inhibitors [136]. A small amount of the labelled ligand is first bound to protein A and is subsequently displaced by titrating the unlabelled protein B. In that way, the inhibition constant *K*_i_ of the unlabelled ligand can be measured. Since the comparison is always of the *K*_i_ of the unlabelled inhibitor, the labelled one does not have to be physiological; therefore, any adverse effects that might appear in this system become unimportant. Since the IC_50_ is the concentration of inhibitor necessary to displace half the labelled ligand, if [A*_t_*] ≪ *K*_d_, IC_50_ is related to *K*_i_ by3.14
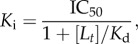
where [*L_t_*] is the concentration of the labelled ligand and *K*_d_ is the equilibrium dissociation constant. For the determination of absolute affinities, measurement of the concentration of the labelled ligand is essential. Such methods, which fall into the category of spectroscopic methods, are very useful because additional information can be derived, such as structural data, binding distances between the fluorophore and the protein, etc. However, these are successful mostly for high-affinity interactions and are limited in studying more complicated equilibria.

This is mainly because the response is not a direct measure of binding, but rather proportional to it [133]. Overall, measurement of an affinity value for protein–protein complexes is always associated with the method used and the experimental conditions reported. For example, FP assays are homogeneous assays that give robust results if the size ratio between components of the complex is high [139]. For complexes of different natures, measurements are performed under different temperature, ionic strength and pH. These differences could lead to an observable variation over the reported data. *K*_d_ values are usually reported with standard errors of 20–50%, equivalent to 0.1–0.25 kcal mol^−1^ for *ΔG*_d_ [102]. Changes in temperature (18–35°C) or pH (5.5–8.5) can alter *K*_d_ by a factor of 2 or 10, respectively, corresponding to 0.3–1 in a logarithmic scale. In addition, the stoichiometry of the interaction (*n*) can be determined with a precision of ±20%, as reported by Wilkinson [133]. Moreover, incorrect corrections for non-specific binding, usage of a labelling method for proteins that may alter the binding behaviour of the complex, presence of non-binding contaminants or of contaminants that might enhance binding, etc. might also hamper the actual calculation of binding affinity. All these potential sources of errors must be treated carefully during measurement [140].

### 3.2. Conceptual models for biomolecular recognition

**Figure 6.**
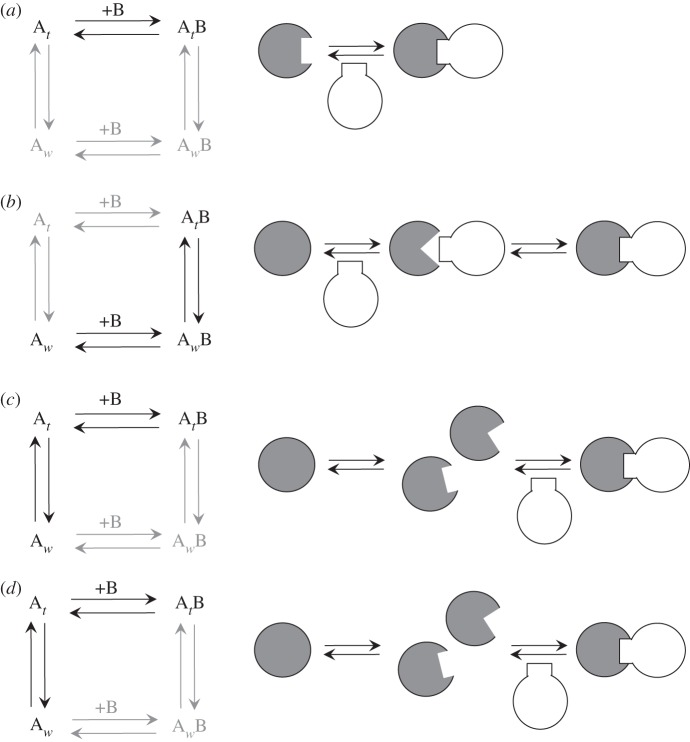
The three basic mechanisms proposed for molecular recognition: (*a*) lock and key, (*b*) induced fit, and (*c*) conformational selection (dynamic fit). On the left, A*_t_* and A*_w_* denote protein A in its tight (binding competent) and weak (binding incompetent) conformation. The chemical pathways that do not exist in each proposed model are indicated by light grey arrows and the way the binding occurs by black arrows. Note that protein B can also undergo conformational transitions; it is shown here rigid for simplicity.

Since molecular recognition is a fundamental phenomenon governing all processes of life, different models that conceptually describe the process have been developed over the last 130 years [141–146]. Three of the proposed mechanisms to describe binding are shown in figure 6*a*.

For proteins that interact in a rather rigid manner, a lock-and-key binding might occur [141], as hypothesized in 1894 by Emil Fischer [143]. The complex of trypsin with BPTI [27] is an example of such a lock-and-key mechanism: the interface of the unbound structures is nearly identical to that of their bound conformation (interface root mean square deviation (i-r.m.s.d.) is less than 0.3 Å). These interactions, along with other examples found in the literature [102], show that one plausible mechanism for protein binding is that one protein might be a (near) rigid complementary image of its partner protein.

A second mechanism describing molecular recognition is the induced-fit model, proposed by Koshland [142,147]. In induced fit, binding of one protein to the other induces specific conformational changes that result in the bound complex (figure 6*b*). The induced-fit model describes that:

— a precise orientation of catalytic groups is required for the reaction,
— proteins might cause an observable change in their binding interface, ranging from small side-chain or surface loop movements to large hinge movement of domains or even folding/unfolding events, and
— these changes will bring catalytic groups into the proper orientation.

**Figure 7.**
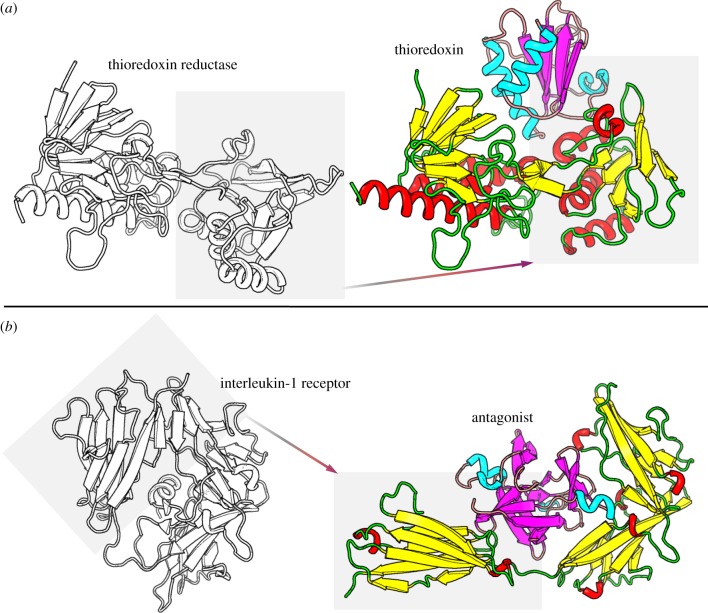
Conformational changes in protein–protein complexes; unbound conformations are shown in greyscale, whereas bound conformations are shown in colour code by assigning a secondary structure; (*a*) the complex between thioredoxin reductase and thioredoxin is illustrated in cartoon representation, and (*b*) the interleukin-1 receptor in complex with its antagonist; both complexes undergo extensive conformational changes upon ligand binding (see also text).

A third mechanism of molecular recognition is the fluctuation (dynamic) fit [143] (figure 6*c*), also recently rediscovered and termed (among others) conformational selection [148,149], conformational selectivity [149], population shift [150], selected fit [151] and pre-existing equilibrium [152]. For consistency with current literature, the conformational selection term will be used here. The conformational selection model hypothesizes that the reactants pre-exist in multiple conformations, the best fitting one of which will proceed to form the product complex. Conformational selection has been reviewed by both Koshland & Neet [153] and Citri [154] considering that it is either a useful addition to the induced-fit hypothesis or an alternative mechanism of macromolecular recognition: fluctuating protein molecules (the concept of protein motility) could provide a good basis for the conformational changes that occur during recognition, where one particular form that is able to bind the substrate will further proceed to react. Conformational selection has been observed in several macromolecular recognition events, even coupled with the induced-fit model [155–158], both in a simultaneous [157] and in a sequential manner [158]. Simultaneous occurrence of both mechanisms means that, depending on ligand concentrations, a shift in the recognition mechanism is observed. Hammes *et al.* [157] observed that at low ligand concentrations conformational selection dominates the binding process, whereas, by increasing the concentration, an induced-fit mechanism is observed. Sequential occurrence of both processes simply implies that the conformation selected from the fluctuating biomolecules undergoes a subsequent structural rearrangement in the intermediate complex that then proceeds to the final bound form [158,159]. Although a clear distinction between induced-fit and conformational selection is hard to observe experimentally, both can be equally plausible for observed conformational changes. Conformational changes are illustrated here for the thioredoxin reductase–thioredoxin complex (PDB ID: 1F6M): thioredoxin undergoes a conformational change of 6 Å in backbone r.m.s.d., whereas the interface of the proteins differs by almost 5 Å, a result of a rotation of the nucleotide-binding domain by 67° (figure 7*a*). A more notable example is the complex formed between the antagonist of the interleukin-1 receptor and its receptor: when the receptor molecule is in its unbound conformation, its globular shape is maintained but the binding site is hindered by its C-terminal domain with which it strongly interacts. However, in the bound conformation of the complex, the C-terminal domain is displaced following a hinge motion, allowing the antagonist to bind in the active site. This motion results in an r.m.s.d. of the receptor molecule's backbone as large as 20 Å (figure 7*b*).

The concept of allostery, as originally proposed by Monod *et al*. [144], also falls into the conformational selection mechanism for molecular recognition. It states that proteins may exist in discrete interconvertible states independent of the ligand structure and/or occupancy; the ratio of these different conformational states is determined by the thermal equilibrium. Presence of ligand merely shifts the equilibrium toward one state or another. This model quantified allosteric events and provided the thermodynamic basis for the dynamic-fit model, elaborated by Burgen [146] and others.

Clarification of which model prevails in macromolecular recognition has not yet been provided since all three distinct conceptual models have been observed experimentally. As a general scheme, one should bear in mind that all three mechanisms may exist both in a simultaneous or in a sequential manner, being recognition mechanisms that can cover a broad spectrum of binding events [157,158,160].

### 3.3. Overall determinants for binding affinity

Various structural determinants of the binding affinity of protein–protein complexes have been proposed throughout the years leading to the construction of different models [28,126,138,161–174], covering nearly all physico-chemical aspects of both the reactants and the product complex. All descriptors for binding affinity must meet four criteria in order to be related to binding affinity:

— They themselves, or their indirect/direct physical effects, must be generated in the complex structure and be absent or different in the unbound conformation of the reactant proteins. If this descriptor or its effect is always constant (its value does not change) between the free and bound forms of the proteins, it must not have any impact on binding affinity else the definition of binding affinity (see §3) will be violated.
— Descriptors that are related to the association of the complex are describing the *k*_on_ rate. Since the *k*_on_ rate is concentration dependent, at least one of the descriptors must also be concentration dependent.
— Descriptors related to the *k*_off_ and, therefore, the dissociation rate of the protein–protein complex must not be concentration dependent, since otherwise the definition of binding affinity would again be violated.
— Descriptors must be causal, since the observation of a correlation does not necessarily imply causality.

#### 3.3.1. Buried surface area

The BSA has been the primary descriptor to be related to binding affinity, and more specifically to the intrinsic bond (or interaction) energy, *ΔG*_bond_, according to the Chothia–Janin model [28]. Further justification has been provided by Miller *et al.* [175], who showed that BSA compensates for the area not buried intramolecularly within the potentially unstable subunits.

BSA is a macroscopic descriptor for the hydrophobic interactions of proteins and its magnitude has been estimated to be 0.025 kcal mol^−1^ per 1 Å^2^ of hydrophobic surface removed from contact with water,3.15
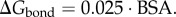


**Figure 8.**
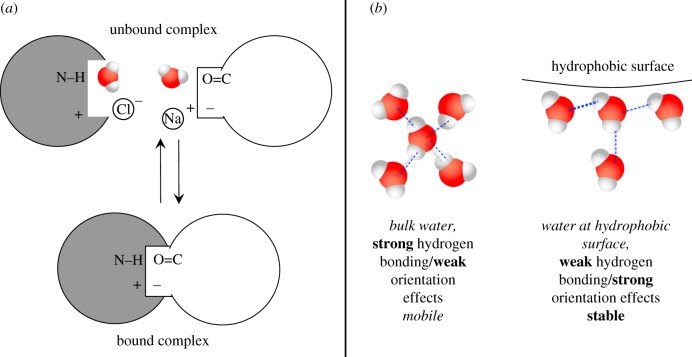
Water in protein–protein interactions and the explanation of the Chothia–Janin theory for the affinity of protein–protein complexes; (*a*) intermolecular interactions are recovered in the bound conformation, being already present with the molecules of the solvent and its ions; (*b*) water at hydrophobic interfaces loses its entropy in comparison with bulk water, which is highly mobile.

This hydrophobic interaction is not only a favourable attraction of hydrophobic surfaces, but also expresses the gain in entropy of the water molecules released upon complexation (figure 8*a*). Since water molecules are less mobile near hydrophobic regions in the reactants, when the product complex is formed, water molecules will be released into the bulk solvent and gain mobility, and thus entropy (figure 8*b*). All other non-covalent interactions observed in the interface are theorized as negligible, since proteins are never in vacuum, but are highly solvated when unbound (figure 8*a*).

Therefore, all interactions of an interface are always satisfied, in both the unbound and bound conformations of the proteins, by contacting solvent molecules or protein residues, respectively. This model however neglects, for example, salt bridges or cation–π interactions, because, even if counter-ions are present, the strength of the interaction might vary depending on the nature of the ion. Despite that, the Chothia–Janin model makes clear that the net contribution of non-covalent interactions, even if zero, must not be ignored because interactions determine the specificity of the complex. A highly specific interaction must reconcile with three criteria, all concerning interface complementarity:

— *Complementarity of ions*. If not all charged groups form salt bridges in the interface, the subunit association would require an ionic bond to the solvent (2–6 kcal mol^−1^) to be broken and, therefore, would highly destabilize the protein–protein complex.
— *Complementarity of hydrogen bonds*. A hydrogen bond that is not satisfied within the protein–protein interface would result in a large change in free energy (0.5–6 kcal mol^−1^) [176].
— *Steric complementarity*. Although van der Waals interactions are weak in nature, the number of atoms in the interface is large, and therefore they contribute to the specificity in a non-negligible manner.

The contribution of macroscopic descriptors of hydrophobic interaction (BSA, apolar BSA, polar BSA, number of atoms in the interface, etc.) to the binding affinity has been validated in a qualitative manner for a large number of complexes assembled [102,177]. For complexes that bind without obvious conformational change, these descriptors exhibit very significant relations to binding affinity, in an, almost, linear manner [102]. On the other hand, the affinities of complexes that undergo conformational changes are not in agreement with the Chothia–Janin theory [102]; therefore, hydrophobic interactions [28] must not be the only determinant for the intrinsic bond energy.

#### 3.3.2. Hot spots and anchor residues

Warm- and hot-spot residues represent only a small fraction of interface, yet these residues contribute significantly to the binding free energy [161]. Warm and hot spots are defined as the residues whose mutation to alanine results in a destabilization of the bound state ensemble by 1–2 and 4 or more kcal mol^−1^, respectively. Null spots, in contrast, do not generate such a free energy difference. Experimentally, the contribution of a residue to the binding free energy can be assessed via alanine scanning mutagenesis, initially described by the Wells group [178,179]. A mutation to alanine essentially removes the side chain of the reference residue, leaving only the β-carbon. Subsequent kinetics analyses may provide clues regarding the role played by individual residues in protein binding. Note that a mutation to glycine might theoretically be a better solution because the whole side chain is removed. Nevertheless, mutations to glycine are not preferred as they might introduce local or global changes to the conformation (and dynamics) of the molecule.

Several algorithms have been developed [180–185] to identify hot-spot residues on protein–protein interfaces; these have been recently extensively reviewed [186–188]. Although they can be classified into two general classes (energy-based and feature-based methods), all are built on the following observations for the hot spots:

— They are most often found in central regions of the interface [161].
— Their amino acid composition differs from that of non-hot-spot residues [182].
— They are more conserved than non-hot spots [189].
— They are occluded from solvent [161,190].

Subsequently, the ‘water exclusion hypothesis’ (or O-ring theory [161]) has been proposed that may rationalize the role of the hot spots, whereas coupling of hot spots has also been reported [191]. Briefly, hot spots that are buried in the interface are surrounded by polar regions of higher packing density. These regions occlude solvent and lower the local dielectric constant and consequently enhance the effect of dipole–dipole or ionic interactions in the formed complex [161,190]. Li & Liu [192] have also hypothesized a double water exclusion hypothesis, where hot spots are always water-free.

Hot-spot residues clearly demonstrate that hydrophobic interactions are not the absolute determinant for binding as described by Chothia and Janin. It is evident that the three complementarity principles mentioned above can be violated. Still, the hot-spot theory is qualitatively in line [190,193] with the Chothia–Janin theory [28] because bulkier residues tend to be found more frequently in hot spots, and these have the largest surface area [194].

Hot spots can affect either *k*_on_ or *k*_off_ (or both) [195], suggesting that the kinetic behaviour of the complex is affected in a different manner by specific hot spots. As an example, mutation of Arg^17^ to Ala in the trypsin—PTI complex leads to a significant effect on both *k*_on_ and *k*_off_ rates, whereas Lys^15^ to Ala has only a marginal effect on *k*_on_ but a similar destabilization effect on *k*_off_ to the Arg^17^ to Ala mutation [196]. The Camacho group has proposed that amino acids that bury the largest solvent-accessible surface area after forming the complex have anchor side chains that are found in the free form in conformations similar to those observed in the bound complex [162]. Such anchors are proposed to reduce the number of possible binding pathways and therefore avoid structural rearrangements at the core of the binding interface. This would allow for a relatively smooth recognition process. Anchor residues must provide most of the specificity necessary for protein–protein recognition [196], whereas other important residues on the interface contribute to the stabilization (and, therefore, the off rate) of the formed complex [196]. Although the observed anchor residues can rationalize encounter complex selection, the transition from the recognition state to the final complex structure is difficult to determine computationally because of the increasing role of short-range interactions that may be harder to evaluate. In general, despite the fact that hot-spot residues are found in protein–protein interfaces, all evidence for their existence comes primarily from rigid and tight protein–protein interactions. This remains to be experimentally explored for transient complexes and complexes showing large conformational changes upon binding in particular [197].

#### 3.3.3. Allosteric regulators and non-interface affinity modifiers

Although allostery has been defined initially as the regulation of a protein by a small molecule that differs from its substrate [144], the definition changed to account for regulation of a protein by a change in its tertiary structure/QS induced by a small molecule. In general, allosteric effects are now recognized as changes in the dynamics or structure of a protein by a modulator; the latter can be of any type, from a small molecule to another protein [198]. Such changes can shift the population of the inactive protein to its active form, thereby significantly altering its binding affinity, e.g. the binding of oxygen to haemoglobin. Examples of such ligands can be, besides oxygen, electron donor organic molecules (e.g. ATP), or post-translational modification events, such as phosphorylation, the latter being the most common covalent protein modification to achieve allosteric control. Such modifications alter the binding affinity of the partners through changes in the dynamics and/or structure of the chains that interact. Therefore, not only interfacial or rim regions can affect the binding affinity of protein–protein interactions, but also modifications of sites remote from the interfacial region through any possible mechanism of allosteric regulation.

## 4. Structure prediction of macromolecular complexes: is the docking problem still unsolved?

Although current structural biology tools have broadened our knowledge in single protein structure, function and dynamics, the situation differs substantially in the case of protein–protein complexes: owing to experimental limitations in probing protein–protein interactions [199] and solving the structure of biomolecular complexes [200] complementary computational approaches are often needed to assist experimentalists in investigating how two proteins of known structure interact and form a three-dimensional complex.

Protein–protein docking algorithms have been developed for this purpose. They use geometric, steric and energetic criteria to predict the atomic structure of a complex [64,201,202]. Every docking program incorporates two key elements:

— the search algorithm that samples configurational and conformational degrees of freedom and
— the scoring function that ranks the generated solutions.

Although predicting the structure of a complex by docking should be relatively simple for proteins that bind with near-rigid body manner and have highly complementary interface regions (such as trypsin–PTI [27] or barnase–barstar [115]), this is clearly not the case. Finding a correct solution for a biomolecular interaction at atomic resolution can be influenced by several factors inherent to any simulation of biomolecular recognition:

— Proteins are not static structures, as explained in §3.2. Their highly dynamic nature can cover the entire scale of conformational changes upon binding from small side-chain reorientations to unfolding/folding transitions. Next to that, different motions of the protein molecules can be exhibited in solution, such as hinge motions [203], secondary structure rearrangement [204], or even high plasticity of the interfacial region [205]. Although several methods can be used for predicting protein dynamics and/or conformational changes [206], none has been shown to perform reasonably well for proteins with different motions [207]. For example, protein motions can be experimentally monitored within a time scale of femtoseconds (e.g. with neutron scattering) to more than a second (e.g. with SAXS, SANS or H-D exchange), whereas molecular dynamics simulations can reach up to milliseconds (but not in a routine manner, being rather limited to nanoseconds–microseconds in most cases) for systems of small to medium size [208].
— The binding site is not always conserved or cannot always be identified [209–211]. Again, results show that most recent interface predictors can distinguish an interface with fair accuracy [188]. However, for weak transient protein–protein complexes, interface prediction might fail [188,210,211].
— Current docking methods cannot distinguish whether two proteins will bind or not, i.e. predict the binding affinity. Docking programs will always yield some answer, independently of the affinity of the protein–protein interaction [212]. Recent studies have highlighted this fact, but, to date, no single docking program has been shown to be successful in identifying native complexes in cross-docking studies, except in the case of highly complementary interfaces [212–215]. Cross-docking is defined as the all-against-all binary docking procedure in which all combinations of proteins are docked to each other and the native complexes must be predicted.
— Scoring, defined as the selection of a preferred solution from the pool of generated conformers, has greatly improved during recent years [215], driven, among others, by blind prediction experiments such as CAPRI [216], the Critical Assessment of PRedicted Interactions (http://www.ebi.ac.uk/msd-srv/capri/). There are even strong critiques about scoring [177,217], even noting that it might be nearly random [218].

Most docking methods are successful for proteins that undergo minor-to-medium conformational rearrangements upon binding. For these systems, scoring functions can identify near-native models that can be subsequently refined [219,220]. Next to that, implementation of novel clustering algorithms [221–223] (clustering refers to the identification and classification of similar docking predictions into clusters) is allowing more efficient analysis of similar solutions, reducing both the computational time and the heterogeneity that could hinder identification of near-native poses.

Recently, there has been a trend in docking simulations to incorporate available experimental information into the docking and/or scoring process. This can dramatically reduce the conformational space to be sampled [202,224,225]. Such information can be used either *a priori* in docking, and therefore drive the docking procedure [226,227], as was originally done in HADDOCK [224], or *a posteriori*, meaning that generated solutions are filtered according to the experimentally observed attributes of the complex [225,228,229]. Recently, more groups are integrating experimental data coming from different sources and the idea of integrative docking [230,231], originally described in the initial HADDOCK publication [224], has become a matter of great importance in current molecular modelling research [232,233]. Integrative docking can be used either for modelling large macromolecular complexes [234], such as the nuclear pore [235] or other cellular machineries, using for example experimental data such as electron density maps [236], or for the detailed characterization of macromolecular assemblies of lower molecular weight using rather classical experimental information from NMR [224]. As an example, approximately 100 biomolecular structures of complexes determined using HADDOCK [236] in combination with various amounts of experimental data (mainly NMR) have been deposited into the PDB [15] as of November 2012.

Although docking is a powerful technique to predict the structure of a complex, based on its known constituents, prediction of the complex based on homology, the so-called template-based methods, is now rapidly increasing [237–239], as illustrated by novel theoretical applications [211]. The Vakser group has recently claimed [211] that templates exist for nearly all complexes of structurally characterized proteins in the PDB, although the authors also report that such observations have not been validated for targets released during the CAPRI experiment. Also, Barry Honig's group has already shown that homologous interfaces can be identified for a vast number of protein–protein complexes and that the expected interface should, in principle, look similar to related ones that have been crystallographically determined [210]. This is, however, not always the case [240,241]. For example, the exact interaction geometry is less likely to be conserved as illustrated by the homologous complexes of the chemotaxis histidine kinase CheA with its phosphorylation target CheY for *Escherichia coli* and *Thermotoga maritima* [242]: in this system, a rotation of approximately 90° is observed between the formed interfaces [242]. In general, however, close homologues (30–40% or higher sequence identity) have been shown to interact in a rather similar manner [243,244].

### 4.1. Is scoring in protein–protein docking related to binding affinity?

Several models have been developed to date for predicting the energetics of macromolecular complexes [28,126,138,161–173,245]. Although some have been very successful on small training sets [126,163], and even coupled to successful docking predictions [246,247], the published models did far less well on larger datasets [168,169,177] and their predictive value remains, in general, poor [177].

For algorithms developed for protein–protein docking coupled with binding affinity prediction, the classical model of Horton & Lewis [126], aimed at predicting binding affinity by decomposing the interface into its polar and apolar BSA, showed a very strong correlation with experimental measurements and crystal structures that were available at the time it was developed [126]. Nowadays, this model is clearly insufficient for binding affinity prediction, since the BSA is moderately correlated with the binding affinity, even for rigid binders (*r* = 0.54 for 70 complexes) [102]. Another example is the algorithm based on the Freire equations [245] for describing binding free energy and modified for predicting binding affinity of a protein–peptide interaction by the Holmes group [247]. The algorithm did fairly well in predicting the actual energy of the reference structure even when coupled with docking; however, a lot of non-native poses generated had equivalent binding affinities, a common problem. The Holmes function assumes that the complex binds without any conformational change [245]. This contrasts with the current view of protein–peptide recognition indicating that, next to the multitude of conformations that a peptide can adopt in solution, folding events occasionally happen upon binding [61]. Another binding affinity predictor coupled with docking is the one developed by Ma and co-workers [165]. Their function ranked and scored the docking results for 10 protein complexes and, while it showed encouraging results, it did not succeed in ranking native solutions first [165]. As far as scoring functions in protein–ligand docking are concerned, these have been optimized mainly for drug design purposes. This means that an estimate of the binding affinity of the ligand can be obtained only in a qualitative and relative manner and for structurally similar ligands. In contrast, protein–protein docking scoring functions have not been developed for predicting binding affinities [177], but rather for identifying the best solutions. Top-performing scoring functions in protein–protein docking [224,248–250] have proven to be reasonably reliable against blind cases in the CAPRI experiment [216,251], being able to identify models close to the experimentally determined ones. However, the same functions poorly predict experimentally measured binding affinities [177]. Next to that, scoring functions are not yet able to distinguish binders from non-binders, as shown by cross-docking simulations. A large-scale effort to predict designed interfaces that do actually bind was made by 28 different groups in a recent CAPRI experiment [168]. Results show that the algorithms can efficiently distinguish binders corresponding to experimentally determined structures from non-binders with designed interfaces. However, all scoring functions failed to predict the designed interface that actually binds from the remaining designs (86 in total) that do not.

### 4.2. Structure–affinity models for protein–protein binding affinity prediction

Various sophisticated approaches for estimating the affinity of protein–protein interactions have been developed to date [252], some of which also include elaborate models that approximate the energetic contributions of the solvent [253]. However, in the context of macromolecular docking, where thousands of models may be generated, these methods are computationally prohibitive. Alternative, more approximate methods that mostly relate to changes in the solvent-accessible surface area upon binding have been proposed instead and these will be discussed in the following.

Since the initial model of Chothia & Janin [28] for predicting the interaction energy of protein–protein complexes, an extensive binding affinity benchmark has been assembled [102]. This dataset includes 144 protein–protein complexes of different affinities and amount of conformational changes to serve as a catalyst for coupling docking results to binding affinity prediction, or just for deriving new binding affinity predictors. Three original algorithms have been developed to date using this benchmark [169,170,172].

One has been developed using descriptors covering all possible combinations of residues in the interface for different binding conformations of the complexes (840 descriptors for 144 complexes in total) [172]. Using a genetic algorithm, these descriptors could be reduced to 378, most of which describe hydrophobic and steric interactions. This number is still much higher than the number of experimental data, indicating possible over-fitting.

Moal *et al.* [169] have designed a machine learning approach, combining four different machine learning methods. Although their results are fairly good for the training set, when the four methods were combined using a consensus approach, they yielded a correlation coefficient *r* with experimental measurements of 0.55, similar to the one that the simple BSA shares with the affinity of rigid complexes [102]. Another multiple regression model from the Weng group [170] exhibits a slightly higher correlation (*r* = 0.63). However, the predictive power for affinities of antibody–antigen complexes is insignificant (*r* = 0.24). Both methods cross-validated their algorithms using leave-one-out-cross-validation (LOOCV). The idea behind this cross-validation method is to predict the affinity of a single protein–protein complex from the dataset, based on the optimized regression equation derived from all other complexes. There are some concerns about LOOCV:

— It tends to include unnecessary components in the model and has been shown [254] to be asymptotically incorrect.
— It does not work well for data with strong clusterization [255].
— It underestimates the true predictive error [256].

The Weng group used all the data for training [170]. No independent test set for validating the model was assembled. The model developed by Moal *et al.* [169], who did use an independent test set, did not hold any predictive capacity on this test set.

#### 4.2.1. Possible reasons for the limitations of current scoring and affinity prediction models: is there a theoretical prediction limit?

Different possibilities can account for the poor prediction of binding affinities using current biophysical models:

— The quality of the experimental data or the crystal coordinates might be ambiguous.
— Very few, if any, of the present models do account for conformational changes taking place upon binding or for the presence of cofactors that might be needed for binding.
— Allosteric regulation or more complicated kinetics of the complex (two-state kinetics, etc.) might hinder actual predictions. Current models only account for the simplest of the mechanisms—the lock-and-key model, as described in §3.
— Effects of pH, temperature, concentration and solvent are usually ignored.
— The performance (especially for affinity prediction models) depends on the quality and size of the set of experimental data used for testing, as well as on the diversity of the biological systems they represent.
— The current models only account for properties of the interface [102], or, rarely, from the rim region—the latter, if included, only for *k*_on_ prediction [138,257]. None account for contributions from the non-interface surface, which can play a significant role in modulating affinity (see §3.3).
— A final possibility is that linking a structure that has been determined in its crystalline state with the affinity that has been measured in solution state can introduce ambiguities in the derived results because of the different natures of the two states.

Overall, the ideal prediction limit that can be set for structure–affinity models (assuming that all modelling ambiguities are eliminated and results are only dependent on the measured data) must be within the experimental error, which, for a large dataset, can change *K*_d_ by a factor of 10–50, and *ΔG*_d_ by 1.4–2.3 kcal mol^−1^ [102,172].

**Figure 9.**
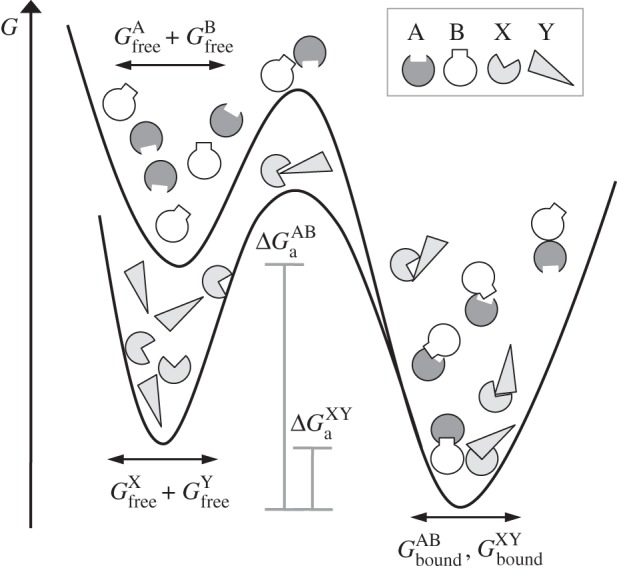
Schematic of the energy landscape of two different protein–protein complexes.

Finally, one of the central reasons for current models' limitations could well be that the current scoring functions do not account for the underlying energetics of the free components. Figure 9 illustrates this point: assuming two different protein–protein complexes, A–B and X–Y with similar energies of their bound state but different energies of their free states, any model considering only the bound state will predict similar binding affinities for those two complexes4.1
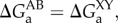
while, experimentally, they will have different affinities owing to the differences in their respective free states,4.2
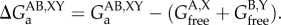

The free state contribution is typically neglected in docking.

While docking scoring functions might not perform well in affinity prediction, this does not imply that they fail in scoring docking poses for which they have been developed. Indeed, most do show a strong performance in ranking and selecting high-quality models in the CAPRI competition [216,251].

An ideal scoring function that could also predict binding affinity should, in principle, be able to (indirectly or directly) account for the free energy of the unbound partners. Some binding affinity prediction algorithms can reasonably well describe the energy of a (near) rigid binding complex [258]. However, predicting the binding affinity of non-rigid binders will require a more detailed statistical–mechanical treatment in which the full ensemble of unbound structures for each partner, and their contribution to the free energy of the free state, should be considered. Such an approach should, in principle, be able to deal with more flexible molecules. A full description of the free energy conformational landscape of highly flexible or even (partially) unfolded molecules will remain out of our reach for the near future.

Overall, models developed to date describe the thermodynamics of an association reaction by its product only, ignoring reactants and possible accompanying structural changes. Novel functions will have to be developed that can predict the dissociation constant within the experimental error in order to have an actual use in modern drug discovery for protein–protein interactions. The availability of a protein–protein binding affinity benchmark [102] should foster the development and improvement of binding affinity prediction algorithms. Hopefully, in the not too distant future, binding affinity prediction and scoring will start to converge.

### 4.3. Prediction of kinetic rates

The association of protein–protein complexes is dictated by the rotational and translational diffusion of the partners, their surface properties, the electrostatic interactions that guide the interaction, as well as the solvent properties, which are, for example, at the origin of the hydrophobic effect. Several simple models have been constructed to predict *k*_on_, mostly based on the Einstein–Stokes equation [259] and Poisson–Boltzmann calculations [260]. Although the limit of collision rate is approximately 10^10^ M^−1^ s^−1^ (calculated by the Einstein–Stokes equation), no single protein–protein complex can achieve this without the aid of electrostatic steering [257]. This limit is three to six orders of magnitude above typically observed association rates, highlighting that most collisions do not lead to fruitful association. Much work has been done on the prediction and improvement of *k*_on_ rates especially for complexes whose association is assisted by charge interactions. Studies have revealed that enhancing electrostatic steering leads to a substantial increase in *k*_on_ [261–263], reaching the limits of the diffusion collision rate. One of the most recent models proposed for predicting association rates is TransComp [138]: it implements the transient-complex theory for predicting *k*_on_ and simulates the formation of a transient complex via diffusion where proteins have near-native separation and relative orientation but have not yet formed short-range interactions. The theory predicts that *k*_on_ is defined as4.3
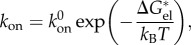
where the basal rate constant for reaching the transient complex by random diffusion is included and the electrostatic interaction free energy of the transient complex. A moderation factor *f* is applied to 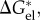 when the latter is very negative, to correct for overestimation of *k*_on_,4.4
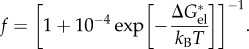


The transition-state theory applied to protein–protein *k*_on_ rate prediction has been tested on 49 protein–protein complexes with known *k*_on_ rates ranging from 2.1 × 10^4^ to 1.3 × 10^9^ M^−1^ s^−1^ [138]. The correlation between the predicted and experimental log *k*_on_ has an *r*^2^ of 0.72, and the r.m.s.d. is 0.73, corresponding to a fivefold error in *k*_on_ prediction.

The method is valid so far for complexes for which the association rate is diffusion limited (*k*_on_ > ∼10^4^ M^−1^ s^−1^) and the reactant proteins undergo negligible, if any, conformational rearrangements.

The Schreiber group developed a *k*_on_ prediction, the PARE function, 13 years ago [264], yielding comparable results to TransComp discussed above (G. Schreiber 2012, personal communication). Briefly, in PARE, *k*_on_ is determined using4.5
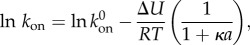
where 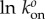 is the basal on rate of the interactions, and the electrostatic and salt influence is explicitly considered; *ΔU* is the electrostatic energy of the interaction, *R* is the gas constant and *T* is the temperature. *α* is set to 6 Å and *κ* is the Debye–Hückel screening parameter relating to the ionic strength of the solution.

*U* in equation (4.5) is calculated using4.6
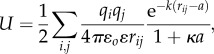
where *i* and *j* are atoms bearing charges and *ɛ* is the dielectric constant of the medium.

*ΔU* is therefore calculated by4.7
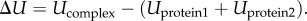


Note that, for proteins that bind with large conformational changes, disordered proteins being at the far end of the spectrum, *k*_on_ determines the binding affinity to a higher extent than *k*_off_ [265], whereas, for rigid complexes, *k*_off_ is the major determinant for binding affinity [265].

**Figure 10.**
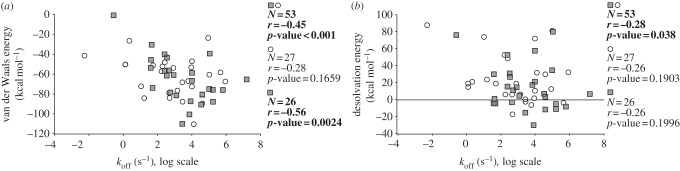
Correlations between some energetic components of the HADDOCK score [217] ((*a*) van der Waals interactions; (*b*) desolvation energy) and experimental *k*_off_ for 54 protein–protein complexes [98,133]. (Near) rigid binders are shown by grey squares, whereas flexible binders are shown by white circles. *r* denotes the correlation coefficient, whereas the *p*-value denotes the corresponding *p*-value (*p*-value < 0.05 is considered significant). Significant correlations are highlighted in bold.

Engineering proteins to achieve desirable kinetic rates is non-trivial [257,263], even in non-crowding conditions [266]. For example, especially for protein–protein binding affinity engineering, charges have been shown to play multiple and complex roles in binding [267,268]. When present in remote areas from the interface, they could lead to the formation of non-specific complexes. For example, Tiemeyer *et al.* [269] showed that the surface charge distribution is very important for the orientation of proteins on lipid membranes. Significant effects of charges on *k*_on_ could be sometimes concomitant with effects on *k*_off_, indicating that the association rate might be difficult to modulate in a significant and controlled manner independently of the dissociation rate [266]. This becomes even more challenging in *in vivo* conditions, where macromolecular crowding can also affect the association rates of protein–protein complexes [123,270–274]: increasing the rates by increasing the effective concentration, and decreasing the rates by decreasing the diffusion of the particles. In recent work, Ando & Skolnick [270] quantified the significant role of hydrodynamic forces in macromolecular motion and Elcock [271] highlighted their importance in protein–protein binding. Another mechanism that can affect *k*_on_ by altering the transition state is the introduction or deletion of steric clashes during association [275]. For example, the association rate constants of the IFN_2–IFNAR2 complex changed when Ala^19^ of IFN_2 (located at the interface) was replaced by a Trp [275]: this mutation introduced a repulsive interaction, resulting in a reduced *k*_on_. However, in parallel, it also reduced *k*_off_, by the formation of a favourable interaction with Trp^100^ on IFNAR2. While substantial progress has been made towards rationalizing the association effects [113,138,171,265,276], dissociation events are still not well understood. For *k*_off_, breaking of short-range interactions between proteins and interfacial properties should be the rate-limiting step. The rate at which the two proteins diffuse away from each other (which will decrease with increased long-range electrostatic interactions) does not seem to affect *k*_off_ much [257]. However, van der Waals interactions only partially correlate with *k*_off_ rates when the dataset of Zhou and the affinity benchmark [98] are considered, and only for near-rigid binders (figure 10*a*). *k*_off_ should also depend on complex–solvent interactions, since in macromolecular crowding conditions hydrodynamic effects are dominant [270,277]. Indeed, a significant correlation is calculated between *k*_off_ and the desolvation energy, independently of the conformational change (figure 10*b*).

Currently, only one model has been proposed to predict the *k*_off_ of protein–protein interactions with reasonable accuracy [173]. The authors provided separate models for predicting *k*_on_, *k*_off_ and *K*_d_, but the properties determining the *K*_d_ are different from those coming from the determinants after division of *k*_off_ by *k*_on_. Also, for calculation of *k*_on_ rates the bound complex was used. This is counterintuitive since *k*_on_ describes the association of the unbound proteins.

## 5. Protein–protein interactions *in vivo*: the p53 example

Protein–protein complexes employ all kinds of attributes that large macromolecules may have in order to accomplish their functions within the cell. *Promiscuity*, *specificity*, *selectivity* and *binding affinity* are factors that can modulate protein–protein recognition and their combination is unique and case specific. These are defined as follows:

— *Specificity* is the ability of a protein to bind a single partner protein for performing a task.
— (*Binding*) *affinity* indicates the existence and strength of an interaction between proteins.
— *Promiscuity* (cross-reactivity/multi-specificity) denotes the ability of a single protein to perform multiple functions, thereby interacting with more than one partner in a specific manner.
— *Selectivity* defines a protein that is binding/using a range of other proteins, but some better than others.

Designing a protein–protein complex with a preferred attribute is very difficult since all these attributes are related to each other [64]. The tumour suppressor protein p53 is a great example for such a combination of properties [278]. p53 is an important hub in multiple signalling networks and is the protein most frequently involved in human cancer. It has been described as ‘the guardian angel of the cell’ [278,279]. p53 has a highly versatile structure, featuring every possible conformation, from ordered secondary structure elements and well-defined folds to completely disordered regions. Its core domain is always folded and binds to DNA and a few other proteins [280], whereas its two flanking regions are mostly in a disordered state undergoing disorder-to-order transitions [280–283]. These may bind hundreds of signalling proteins. A sequence segment within one of these regions exhibits chameleon features [282], meaning that it can adopt three different ordered conformations, excluding loop orientation (α-helix, β-sheet with flanking strands, beta-turn-like), depending on the partner with which it interacts. Therefore, in the cell, interactions of a specific protein binding site with many partners, such as the rigid core domain of p53, are likely to be mutually exclusive, resulting in competition for interactions among alternative partners [281]. Such competition must be a critical determinant for the specificity of the underlying interactions. The selectivity of such binding sites is determined by the relative binding affinities of alternative interaction partners and by the local concentrations of each protein. However, selectivity is extremely difficult to predict, either *in vivo* or *in vitro*, since a number of factors (such as post-translational modifications, subcellular localization and differences in subcellular distributions, interactions with additional proteins) may modify dramatically the interactions.

### 5.1. Cellular complexity, compartmentalization and crowding effects influence protein–protein interactions

As discussed previously, functional, structural and dynamic properties of the individual proteins influence binary interactions. In addition to protein variants coming from post-translational processes [284], alternative splicing [285] and other (e.g. genetic) factors that may influence gene expression [286], the structure of the interactome is one of the crucial factors underlying the complexity of life, from cells to complete organisms. It is highly dynamic with changes as a function of time, localization in the cell, as well as in response to environmental stimuli [287]. Even interactomes from cells derived from the same tissue, or synchronized cells, may substantially differ. As a consequence, a protein that can be found in different cellular compartments may exhibit different functions, different interactions, or discrete post-translational modifications. Therefore, not only the combination of promiscuity, specificity, selectivity and binding affinity for a specific protein–protein interaction defines the recognition but also all the endogenous and exogenous factors that influence the cell.

Protein interactions are governed by several forces, such as compartmentalization and electrostatic and hydrophobic effects. Co-localization, an endogenous property of the living cell, already increases the effective concentration of biomolecules, leading to non-specific protein interactions in their microcompartment. Co-localization, together with normal mechanisms of natural selection, can lead to the formation of interacting domains, hetero- or homodimers [198]. For example, when proteins are not co-localized, a singe mutation can lead to a change of a couple of kcal mol^−1^, but, since the concentration of proteins in the cells is usually in the micromolar to nanomolar range, binding is negligible. However, when proteins are co-localized (boosting greatly the effective concentration of proteins), a small effect on the dissociation constant can translate into substantial binding, since its value can be brought below the effective concentration of the co-localized partner (being approx. micromolar).

One of the most prominent endogenous properties of the cell is macromolecular crowding, a phenomenon that alters the properties of molecules in a solution when high concentrations of macromolecules such as proteins are present [274]. Macromolecular crowding enhances significantly interactions in a non-specific manner and is expected to affect both diffusion-limited and transition-state-limited association reactions, by decreasing or increasing their rates [123,272]. However, it is still unknown to what extent cellular heterogeneity and physiological properties of biological structures are affected, since no single experimental study has yet reported conclusive evidence on the role of macromolecular crowding. Models for macromolecular crowding should be developed in order to have a more realistic view of in-cell protein–protein interactions [271], given the available experimental data [272]. Towards this goal, novel in-cell NMR methodology [288,289] should contribute to our understanding of protein–protein interactions in different cellular environments and under different cellular conditions.

## 6. Design of protein–protein interfaces, modulators and inhibitors

Two general approaches deal with the modulation of protein–protein interactions, namely (i) redesign of the interface by genetic/protein engineering, aiming to alter properties of the protein–protein complex or even the specificity of an interaction, and (ii) inhibitor design, aiming to disrupt protein–protein interactions. A book on this topic has been published by Adler *et al.* [290].

### 6.1. Interface design of protein–protein complexes

Experimentally, redesign of natural protein–protein interfaces has been successfully applied in several cases [64,291], including various systems of structural [292] significance for interface design or major biological importance [293,294]. The current main goals of interface design are to increase the affinity and/or alter the specificity of an interaction [64,295]. Such studies include careful combination of experimental approaches (mutagenesis studies coupled with experimental measurements of affinity and determination of the structure of the complex of the derived variants) and theoretical methods (docking, interface/hot-spot prediction, free energy calculation, calculation of interfacial hydrophobicity, etc.) [292–294,296–300]. Most of the successful design methodologies include either promotion of dipole interactions between α-helices [300–302] or binding of an α-helix to the binding site of the target [293,296,303]. Several other methods have mapped known side-chain interactions from a crystal structure onto another protein that can then be used as a scaffold [304,305]. Recently, homodimer designs composed of paired β-strands have been reported [292,297].

Several limitations of the designed binders have been reported, such as significant rotation of the binders in the crystal structure compared with the predicted orientation of the protein–protein complex, even by as much as 180° [294], or the existence of multiple low-energy binding conformations [296]. It has long been known that introducing changes to increase affinity of the partners might hamper the specificity of the interaction [306]. This may lead to the formation of interfaces with different properties from expected, despite successful engineering of the binders. Another issue regarding interface design is that new hydrogen-bond networks are daunting to design [307], whereas hydrophobic matching of the interface [292] can lead to aggregation. Finally, most of the designs reported to date have been aimed towards protein–protein complexes that have a high degree of surface complementarity. Engineering of more transient protein–protein interactions, with fewer concave interfaces, has not yet been reported. In general, by increasing the affinity of a given protein–protein complex, several other properties of the proteins might be influenced, from their individual stability or solubility to the complex's general properties, such as promiscuity and specificity. Therefore, design of protein–protein interfaces is a daunting task, since careful investigation of all altered properties of the reactants and the derived product should be reported in order to assess the modulation of the interfacial properties in detail.

A very notable example for the limitations of present scoring functions in interface design has been reported [168], where none of the current computational methods used to calculate energetic properties of protein–protein interfaces could discriminate designed complexes that were not able to bind from the one that actually binds. Therefore, although several components of scoring functions should be useful in discriminating designed interfaces from naturally occurring ones, such as the backbone conformational rigidity, electrostatic interactions or solvation energy [168], there is still a gap in our understanding of naturally occurring protein–protein interfaces compared with designed ones.

### 6.2. Small-molecule and peptide inhibitors of protein–protein complexes

The design of inhibitors of protein–protein interactions (protein–protein interaction inhibitors) is also being actively pursued [41,60,197,308–313]. Some designed inhibitors, such as Navitoclax (ABT-263), an inhibitor of the Bcl-2 family of proteins, have even reached pre-clinical or clinical trials [314]. Most of the protein–protein interaction inhibitors target directly the interface of the complexes, the so-called interfacial inhibitors [315]. Note that this is not synonymous with orthosteric inhibitors, which in our understanding bind to the primary active site of an enzyme or ligand binding site of a receptor molecule [316]. Some inhibitors have been developed that bind at remote locations from the interface, preventing conformational changes required for the formation of the complex (allosteric or non-interfacial inhibitors) [315]. Since protein–protein interfaces are larger than classical enzymatic binding sites, inhibitors or modulators that have been designed for these are also larger in size [317]. Therefore, the traditional drug likeness rules set by Lipinski *et al.* [318] are not generally applicable for this class of inhibitors [319]. Properties of inhibitors of protein–protein interactions are still under investigation, although a consensus seems to emerge [320–327]. Rationalization of the chemical space of protein–protein interaction inhibitors by using machine learning strategies or sets of molecular descriptors indicated that commercially available libraries are not sufficiently adequate for targeting protein–protein interactions [328], and their specificity, for example for p53-MDM2 inhibition [329], has not yet been fully elucidated. Current studies indicate that this class of inhibitors is generally lipophilic with a higher unsaturation index and ring complexity than common inhibitors [315]. However, whether lipophilicity is a consequence of the way these inhibitors are designed or of the nature of the interfaces that they target still remains to be explored.

Next to chemical substances aiming at disrupting protein–protein interactions, peptide inhibitors have also been reported [290,308,311,312,315,330]. It has become clear that protein–peptide interactions are of high abundance in the living cell, constituting 15–40% of all interactions [331]. Accordingly, discovery and development of protein–peptide inhibitors is of great interest. A few examples follow considering highly potent and selective cyclic [332,333] and other modified peptides [313,334,335]. Stapled peptides produced by connecting two structurally optimized amino acids have also been reported [336,337]. All have been reviewed recently [338–340]. Peptides that modulate protein–protein interactions may be rationally developed by mimicking one of the two partners involved in a protein–protein interaction [341], or directly derived from the screening of peptide sequences that do not originate from natural proteins [342]. Peptides may be antagonists of protein–protein interactions or inhibit the specified interaction. Examples are also available where the inhibitors shift the protein equilibrium, affecting oligomerization; such inhibitors are termed shiftides [343]. An example is the inhibition of HIV-1-IN by peptides derived from its cellular binding protein, LEDGF/p75 [343]: the derived peptides inhibit HIV-1-IN activity in a non-competitive manner, preventing DNA binding by shifting the HIV-1-IN oligomerization equilibrium towards its inactive tetrameric form rather than the active dimer. Notably, a lead chemical compound, now in clinical trials, ABT-737 [344], has been designed as a peptidomimetic, meaning that modulation of Bcl-XL by the BH3 peptide occurring in the cell was mimicked to derive an inhibitor with similar binding characteristics. However, peptide inhibitors have the disadvantage of being easily degraded and thus not orally available. Also, because of their nature, peptides can interact non-specifically with various targets when present in the cell [345]. Improvements in chemical peptide synthesis are required to allow easy chemical modifications and use of non-natural amino acids [328,338,341], in order to possibly improve the stability and specificity of peptide inhibitors. Structurally improving peptides for binding specificity should also improve their advantage over small molecules as, in principle, owing to their nature, they should be easily accommodated in the interfaces of protein–protein interactions.

## 7. Conclusions

Despite past and current efforts in relating protein structure to binding affinity for protein–protein interactions, the underlying dissociation constants, measured *in vitro*, can still not be reproduced computationally within experimental error for a large dataset of protein–protein complexes. It has been evident that the main physico-chemical measure that relates to binding affinity for protein–protein interactions is the interface area. However, for protein–protein complexes that change significantly their conformation upon binding, even the interface area that is buried upon complexation is not related to binding affinity. Consequently, there must be a significant entropic contribution that will have to be approximated in the future by accounting for structural properties that may be connected to the complexation entropy.

Apart from the direct contributions from the interface that have already been modelled in a satisfactory manner (see §3.3 and 4), vibrational entropy, translational entropy, rotational entropy, conformational flexibility and solvent effects will also have to be accounted for—and, of course, also the effect of the crowded cellular environment. Spolar & Record [346] have attributed the large excess in entropy observed in flexible association to the conformational entropy after entropy decomposition into the abovementioned terms. The availability of an ever-increasing amount of structural and thermodynamic data for protein–protein complexes should stir developments in this research area and hopefully lead to a better understanding of the underlying relations.

Finally, most work so far has been concentrated on binary protein–protein interactions. Molecular associations including multi-component systems, allosteric interactions, multi-state kinetics, or even conformational transitions of membrane proteins are far from being sufficiently well understood to allow the derivation in a systematic manner of useful structure–affinity relations. We foresee that any theoretical modelling of these interactions in the future will have to follow an integrated approach considering the biology, chemistry and physics that underlie protein–protein recognition.
